# In Memory of Dr Jos. P. Logan

**Published:** 1891-08

**Authors:** 


### IN MEMORY OF DR. JOS. P. LOGAN.

Dr. Joseph Payne Logan was born in Botetourt county, Virginia, November, 1821, and died in Atlanta, Georgia, June, 1891. He was educated at Washington College, Lexington, Virginia, and studied medicine at the Virginia Medical College, Richmond, and the University of Pennsylvania, Phildelphia, graduating at the latter in 1841. He practiced medicine in Culpepper county, Va., until 1854, when he moved to Atlanta, Ga. In the latter part of 1861 he was commissioned as a surgeon in the Confederate service, which place he filled with distinction. At the close of the war he resided in Baltimore, Md., and was Professor of the Principles of Medicine in the Washington University. He returned to Atlanta in 1868 and resumed permanent citizenship and practice there. He held many important trusts. He was in 1861 one of the two delegates appointed to urge the Confederate Congress to locate the Confederate Capital at Atlanta. He was an organizer and President, in 1871, of the Atlanta Academy of Medicine; Professor of Physiology in the Atlanta Medical College; a member and President of the Georgia Medical Association; Vice-President of the American Medical Association; appointed by the Governor member of the Georgia State Board of Health ; author of reports on Small Pox and Yellow Fever in Savannah ; member of the first Board to organize the Atlanta Public Schools, and for several years editor of the ATLANTA MEDICAL AND SURGICAL JOURNAL.

Dr. Logan was a great physician, a noble man and genuine Christian. Like the pastor, the doctor deals with the spirit, but unlike him, heals the body, mingling two sacred missions. Dr. Logan blended physical healing and Christian comforting in a marvellous degree. His sympathy was as gentle as his science was skilled. He had a remarkable patience and subtlety in reading ills and an extraordinary grasp of cure. He was a born doctor by mind and temperament. He had natural medical genius. His strong native intuitions in healing were fortified by the largest culture in medical science. He had a face and form that typified his noble nature. He was a strikingly handsome and imposing man of majestic proportions and carriage, and a head and countenance benignant and comely. He was a rare character, strong yet pure, manly and gentle, proud though tender, able but pious. He had an enormous practice in Atlanta, and it is doubtful if any of its citizens ever shared more widely in the sorrows of its good people, was ever linked more closely to its sacred memories or will be more regretted than Dr. Logan. He was a pious member and efficient elder in the Central Presbyterian church. He was a wise and successful business man and the soul of integrity. He was full of charity and public spirit.

He married in 1843 Miss Ann E. Pannell, of Orange county, Va., who died April, 1885 ; and in June, 1887, Miss Alice Clark, of Atlanta, Ga. He leaves one granddaughter, Miss Laura Grant.

---

THE Kentucky State Board of Health has recently adopted the following resolution:

*Resolved*, That the Secretary be instructed to place upon the list of Medical Colleges, whose diplomas are to be certified and endorsed for registration under the laws of this State, only such colleges as shall, after the session of 1891–92, exact of matriculates and graduates a minimum of requirements not less than those required by the American Medical Association.

---

DR. FORDYCE BARKER, of New York City, died on May 30th, at the age of seventy-three. Dr. Barker was one of the ablest and most prominent of the New York profession. He had for many years occupied the Chair of Obstetrics in the Bellevue Hospital Medical College. He made several valuable contributions to medical literature, the best known of which is his classic treatise on Puerperal Diseases.

---

DR. G. FRANK LYDSTON, of Chicago, has been elected Professor of Genito-Urinary and Venereal Diseases in the College of Physicians and Surgeons, Chicago.

